# Morphological inter-limb asymmetry in youth judokas is independent of competitive level and sex

**DOI:** 10.1186/s13102-025-01440-8

**Published:** 2025-11-24

**Authors:** Joachim D’Hondt, Laurent Chapelle, Tijl Lindekens, Peter Clarys

**Affiliations:** Movement and Nutrition for Health and Performance (MOVE) Research Group, Department of Movement and Sport Sciences, Faculty of Physical Education and Physiotherapy, Vrije Universiteit Brussel, Brussels, Belgium

**Keywords:** Dual-energy x-ray absorptiometry, Body composition, Bilateral, Side-to-side difference, Lean mass, Body composition

## Abstract

**Background:**

Inter-limb asymmetry is commonly regarded as a potential limitation for athletic performance and a contributor to injury risk. However, the influence of training status and sex on asymmetry in judokas remains poorly understood. Therefore, this study aimed to: (1) quantify inter-limb asymmetry in lean mass, bone mineral density, bone mineral content, and fat mass, and (2) examine how inter-limb morphological asymmetry varies across competitive levels (national vs. European) and sexes among young competitive judokas.

**Methods:**

Lean mass, bone mineral density, bone mineral content, and fat mass were assessed using Dual-energy X-ray Absorptiometry in 27 male and 21 female competitive judokas aged 13–17 years. Comparisons between limbs were performed using paired-samples t-tests. Differences in asymmetry across competitive levels (national vs. European) and sexes were analyzed using Mann-Whitney U tests.

**Results:**

Inter-limb asymmetry magnitudes ranged from 1.47% to 8.24% across groups. Overall, judokas showed greater values in the dominant limb compared to the non-dominant limb for most measures (*p* ≤ 0. 010). Mann-Whitney U tests identified significant differences in asymmetry between competitive levels only in lower-limb fat mass for both males (*p* = 0.015) and females (*p* = 0.033), and in lower-limb bone mineral content for females (*p* = 0.039). No significant differences in asymmetry were observed between sexes.

**Conclusion:**

While inter-limb differences were present, the magnitude of asymmetry was generally small (≤ 8%) and did not differ significantly between competitive levels or sexes. These findings suggest that reducing morphological asymmetry in judokas may not be a priority, regardless of competition level or sex.

## Introduction

Inter-limb asymmetry refers to measurable differences in function (e.g., strength and agility) or structure (e.g., body composition) between the limbs, commonly categorized as functional and morphological asymmetries, respectively [1–3]. These asymmetries often arise from repeated unilateral movements associated with daily tasks or athletic training [4, 5]. While some degree of asymmetry is considered a natural phenomenon in humans, larger asymmetries have been linked to impaired athletic performance and are often regarded as potential risk factors for injury in various sports [6–9]. For instance, Ojeda-Aravena et al. [8], demonstrated a negative relationship between morphological asymmetry, assessed using Dual-energy X-ray Absorptiometry (DXA), and high-intensity performance in taekwondo athletes (*r* = −0.56 to −0.76, *p* < 0.05), indicating that greater inter-limb differences in body composition may impair high-intensity performance in taekwondo athletes. Additionally, Kozlenia et al. [10] found that physically active women with skeletal muscle mass asymmetry greater than 3.6% exhibited a higher risk of injury.

Over the last decade, there has been growing interest in inter-limb asymmetry across combat sports [8, 11–14]. Judo is a high-intensity, physically demanding combat sport that involves explosive and complex movements such as throws, counter-attacks, and ground control techniques [15]. As a weight-class sport, judo places significant emphasis on body composition, which is closely monitored throughout the competitive season at the whole-body level to optimize performance [16, 17]. However, given that many judo techniques are executed unilaterally and judokas tend to have a preference for one side or personal technique (tokui-waza), side-to-side differences in functional characteristics and morphological adaptations may develop with increasing training load and warrant further investigation [18, 19].

Several studies have attempted to characterize morphological asymmetries in judokas using various measurement tools, though findings vary widely. For example, anthropometric assessments (e.g., wrist breadth and elbow breadth) have shown relatively small inter-limb differences in 28 elite (0.4–1.6%) and sub-elite (0.7–1.6%) female judokas [12]. In contrast, Mala et al. [20] reported substantially higher magnitudes of asymmetry in adolescent judokas using bioelectrical impedance analysis (BIA), particularly in fat mass (FM), with mean asymmetries as high as 17.30% in boys and 23.30% in girls. These contrasting results suggest that while morphological asymmetry might be relevant to athletic performance and injury risk, large discrepancies across studies and morphological characteristics exist, potentially related to differences in accuracy between devices [21, 22]. Consequently, there is a clear need to assess inter-limb asymmetries using accurate and reliable, yet practical techniques.

DXA is a relatively low-cost method that enables segmental body composition assessments and typically demonstrates low coefficients of variation (1–2%), making it a suitable tool for assessing inter-limb asymmetries in athletes [23]. However, to the best of our knowledge, only one study investigated the magnitude of inter-limb asymmetry in judokas using DXA [14]. This study reported asymmetry values ranging from 1.80% to 12.06% among 56 competitive adult judokas competing at the national, European, and world levels, across both sexes. No statistically significant difference in asymmetry was observed across competition levels (*p* = 0.113 to 0.891) or between sexes (*p* = 0.163 to 0.846). These findings contrast partially with those of Krzykala et al. [24], who investigated morphological asymmetry in 618 Polish non-athletic youths with BIA. While no significant sex differences were found in muscle mass asymmetry, they did report significantly higher FM asymmetry in the arms of female participants (*p* < 0.001) and in the legs of male participants (*p* < 0.001 to 0.002). Beyond differences in measurement techniques, variations in maturation between populations may also help explain these discrepancies. Indeed, longitudinal research has demonstrated that inter-limb asymmetry is strongly influenced by biological maturation [25]. For example, Chapelle et al. [25] demonstrated that each one-year increase in maturity offset was associated with significant increases in upper limb asymmetry magnitude for both bone mineral density (BMD) (1.3 ± 2.2%) and bone mineral content (BMC) (0.6 ± 2.4%) in elite youth tennis players. It is important to investigate inter-limb asymmetry in youth judokas during periods of growth, when athletes may be particularly vulnerable to injury [26].

Given the limited body of research on inter-limb asymmetry in youth judokas, and its potential implications for performance and injury risk, further investigations on the magnitude of inter-limb asymmetry in this specific youth population are warranted. Accordingly, the present study aimed to: (1) quantify the extent of inter-limb asymmetry in lean mass (LM), BMD, BMC, and FM, as measured by DXA, in both the upper and lower limbs of youth judokas; and (2) examine whether these asymmetries vary according to competitive levels (national vs. European) and sex (male vs. female). It was hypothesized that the magnitude of inter-limb asymmetry in youth judokas would differ between competition levels and sexes.

## Methods

### Participants

The study included 27 male and 21 female adolescent judokas aged 13–17 years, comprising 28 European-level athletes (57.1% male) and 20 national-level athletes (55.0% male). European-level participants were recruited from a high-performance judo center in Belgium, whereas national-level judokas were selected using convenience sampling methods. Training frequency was determined based on data provided by the national judo federation, indicating that European-level athletes typically trained six times per week, whereas national-level athletes trained approximately twice per week. Participants were excluded if they were pregnant or had medical implants, hardware, or other foreign objects in the area of measurement. A priori power calculations conducted with G*Power (Version 3.1, University of Düsseldorf, Germany) indicated that a sample of 48 judokas would be required to achieve a statistical power of 0.8 with a significance level of 0.05.

### Procedures

#### Basic anthropometry

Stature was measured using a portable stadiometer to the nearest 1 mm (SECA 216, SECA, Germany) and body mass was determined using a precision digital scale (Radwag WLT60/10/X/3, Radwag, Poland), accurate to within 2 g. Body mass index was calculated as body weight (kg) divided by height in meters squared (m²). All measurements were taken with participants wearing minimal clothing, following the guidelines set by the International Society for the Advancement of Kinanthropometry [27]. Since leg length and sitting height were not assessed, it was not possible to determine the maturity offset of the judokas as described by Mirwald et al. [28].

#### Dual-energy X-ray absorptiometry

Segmental body composition at both upper and lower limb levels was evaluated by a qualified researcher using a dual-energy X-ray absorptiometry scanner (Lunar iDXA, GE Healthcare, Madison, WI, USA). To maintain measurement consistency, the device underwent manufacturer-recommended calibration and quality control procedures before each testing session. Participants adhered to a standardized pre-assessment protocol, which involved fasting for a minimum of four hours, abstaining from vigorous exercise for 24 h, and avoiding caffeine and alcohol on the day of the scan. They were also asked to empty their bladder immediately before scanning to reduce fluid-related variability and wore light clothing with no metal items. During scanning, participants lay supine on the scanning table with their arms positioned comfortably at their sides and thumbs facing upward, in accordance with manufacturer instructions. The full-body scan provided segmental data for the upper and lower limbs, including LM, BMD, BMC, and FM.

Inter-limb morphological asymmetries were calculated using the Percentage Difference Method: ((dominant value – non-dominant value)/dominant value) × 100 [25]. The dominant limb was identified as the one with the highest value for the respective body structure, while the non-dominant limb was the one with the lowest value.

#### Statistical analysis

All analyses were conducted using SPSS software (version 29, SPSS Inc., Chicago, IL, USA). Significance was set at *p* < 0.05. The distribution of the data was assessed using the Shapiro–Wilk test, complemented by visual inspection of histograms and Q–Q plots. As the raw DXA scores were normally distributed, paired samples t-tests were employed to compare dominant and non-dominant limbs. In contrast, asymmetry scores did not follow a normal distribution; therefore, non-parametric tests were applied. The Mann–Whitney U test was used to examine differences in asymmetry magnitude between competition levels (European-level vs. national-level) and between sexes (male vs. female). When significant effects were observed, post hoc comparisons were conducted using the Mann–Whitney U test with Bonferroni correction. Effect sizes were calculated using the formula r = Z/√N and interpreted as follows: 0.10–0.30 = small, 0.30–0.50 = medium, and ≥ 0.50 = large [29].

## Results

Table 1 provides a summary of the descriptive characteristics. No significant differences in age, body height, body weight and BMI were observed between the European- and national-level athletes (U = 285.000 to 365.000, *p* = 0.154 to 0.966). In contrast, the European-level judokas had a significantly higher training frequency compared to the national-level judokas (U = 0.000, *p* < 0.001).

**Table 1:** Descriptive characteristics (mean ± standard deviation) of the male and female European-level and national-level judokas

	Males			Females		
	European-level	National-level		European-level	National-level	
*N*	16	11		12	9	
*Demographic information*						
Age (y)	15.9 ± 1.1	15.3 ± 1.3		15.3 ± 1.1	15.6 ± 0.9	
Body height (cm)	172.2 ± 7.8	172.2 ± 6.9		162.8 ± 5.6	165.8 ± 3.3	
Body weight (kg)	66.3 ± 13.1	65.5 ± 10.4		55.1 ± 8.2	60.5 ± 7.5	
BMI (kg/m^2^)	22.2 ± 2.8	22.0 ± 2.8		20.8 ± 2.8	22.0 ± 2.7	
*Sport-specific information*						
Weekly training sessions (#/week)	6.0 ± 0.0	2.0 ± 0.0		6.0 ± 0.0	2.0 ± 0.0	

*BMI* body mass index, *LM* lean mass, *BMD* bone mineral density, *BMC* bone mineral content, *FM* fat mass

All raw DXA scores are presented in Table 2. In both European- and national-level judokas, the dominant limb consistently exhibited significantly higher morphological values than the non-dominant limb. This pattern was observed in both males (European: t = 4.080 to 6.645, *p* < 0.001; national-level: t = 3.724 to 7.521, *p* < 0.001 to 0.004) and females (European: t = 3.086 to 10.214, *p* < 0.001 to 0.010; national-level: t = 3.825 to 7.532, *p* < 0.001 to 0.005), except for upper-limb BMD in European-level male (t = 0.054, *p* = 0.479) and female (t = − 0.275, *p* = 0.788) judokas, where no significant differences were found.

**Table 2:** Average raw scores ± standard deviation for the dominant and non-dominant upper and lower limb according to competition level

	European-level		National-level	
	Dominant	Non-dominant	Dominant	Non-dominant
Male Judokas				
LM – UL (g)	**3720.5 ± 777.5**	3563.3 ± 754.2	**3324.3 ± 661.6**	3164.1 ± 666.1
LM – LL (g)	**9348.4 ± 1835.4**	9030.3 ± 1815.4	**8710.8 ± 1495.7**	8460.5 ± 1471.4
BMD – UL (g/cm^2^)	0.873 ± 0.244	0.871 ± 0.115	**0.967 ± 0.139**	0.852 ± 0.291
BMD – LL (g/cm^2^)	**1.418 ± 0.194**	1.379 ± 0.184	**1.380 ± 0.155**	1.349 ± 0.148
BMC - UL (g)	**259.4 ± 60.3**	241.6 ± 55.3	**228.5 ± 52.7**	210.5 ± 51.3
BMC – LL (g)	**607.0 ± 146.7**	594.1 ± 141.9	**549.1 ± 99.4**	536.5 ± 94.3
FM – UL (g)	**637.6 ± 208.2**	585.5 ± 191.0	**660.5 ± 208.4**	609.3 ± 194.1
FM – LL (g)	**2004.4 ± 858.0**	1884.3 ± 832.2	**2113.2 ± 786.2**	2054.4 ± 760.2
**Female judokas**				
LM – UL (g)	**2349.0 ± 354.1**	2238.9 ± 346.4	**2458.2 ± 340.2**	2318.4 ± 296.6
LM – LL (g)	**6800.5 ± 800.9**	6529.8 ± 735.9	**7260.9 ± 872.2**	7069.2 ± 915.3
BMD – UL (g/cm^2^)	0.739 ± 0.215	0.756 ± 0.680	**0.907 ± 0.090**	0.855 ± 0.096
BMD – LL (g/cm^2^)	**1.265 ± 0.081**	1.241 ± 0.085	**1.239 ± 0.096**	1.220 ± 0.091
BMC - UL (g)	**180.8 ± 22.9**	169.3 ± 24.7	**179.2 ± 21.0**	166.3 ± 16.8
BMC – LL (g)	**441.3 ± 50.7**	434.4 ± 48.4	**460.6 ± 34.6**	446.1 ± 37.6
FM – UL (g)	**816.1 ± 243.0**	780.0 ± 259.0	**972.4 ± 201.1**	898.9 ± 190.9
FM – LL (g)	**2726.3 ± 683.4**	2618.3 ± 637.4	**3348.3 ± 511.7**	3139.6 ± 510.5

*UL* upper-limb, *LL* lower-limb, *LM* lean mass, *BMD* bone mineral density, *BMC* bone mineral content, *FM* fat mass

significantly higher (*p* < 0.05) dominant values compared to non-dominant values are bolded

**Fig. 1:**
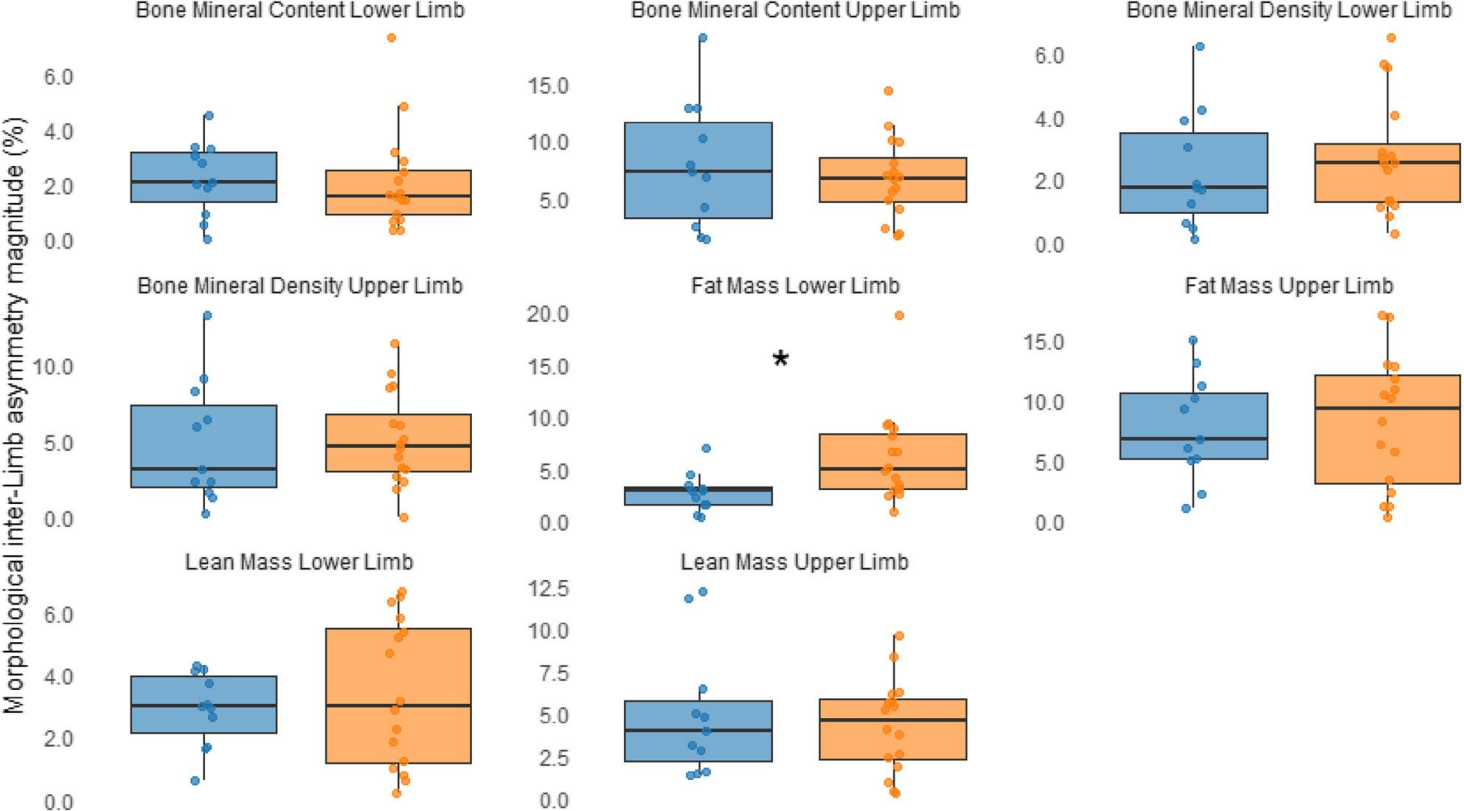
Morphological asymmetry in upper and lower limbs of male national- and European-level judokas, with significant differences indicated by an asterisk. European-level athletes are shown in orange, and national-level athletes in blue

**Fig. 2:**
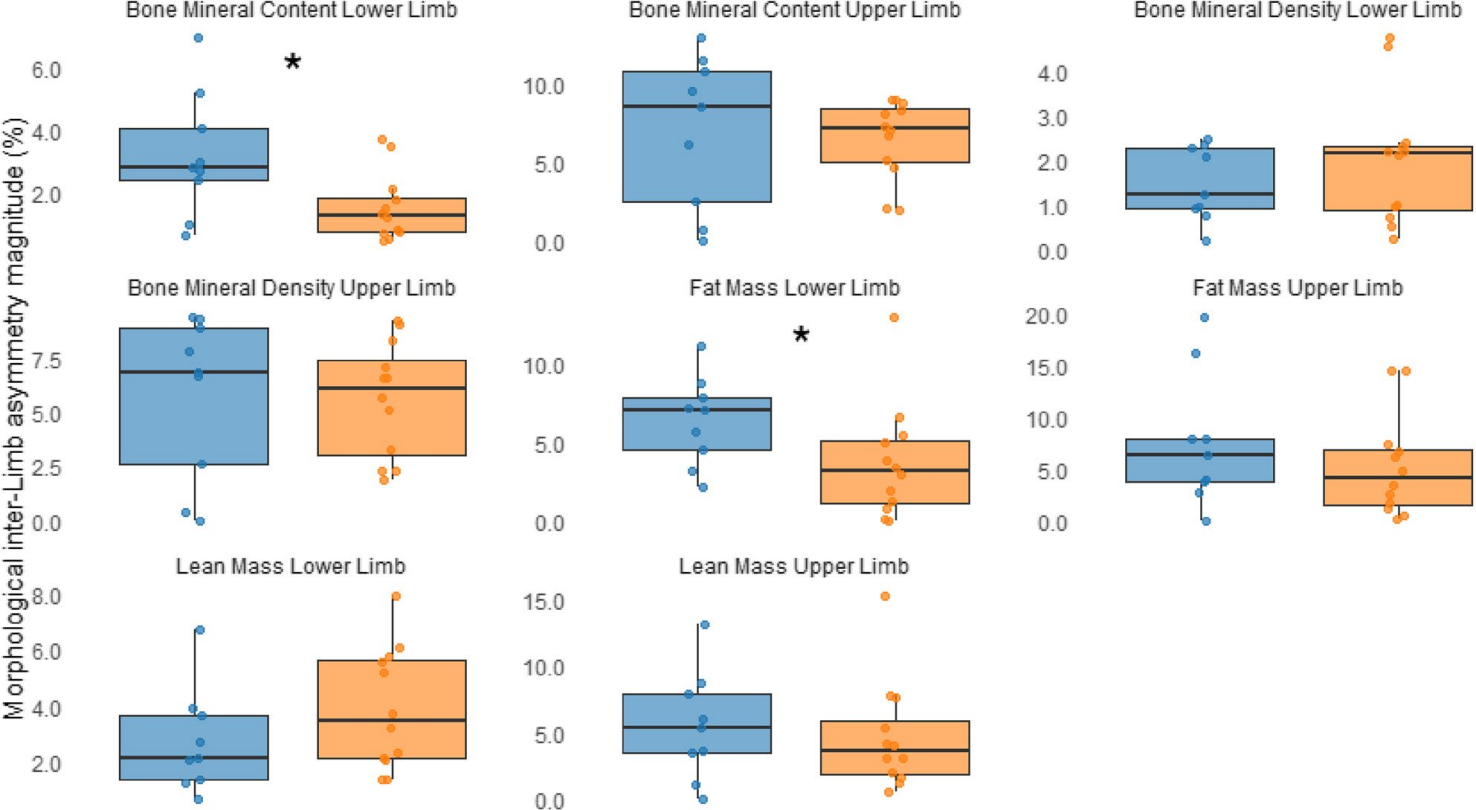
Morphological asymmetry in upper and lower limbs of female national- and European-level judokas, with significant differences indicated by an asterisk. European-level athletes are shown in orange, and national-level athletes in blue

Mann–Whitney U test results indicating differences in the magnitude of inter-limb asymmetry between competition levels are presented in Table 3; Figs. 1 and 2. Overall, no significant differences in morphological asymmetries were observed between competition levels for either sex (males: U = 73.00 to 86.00, *p* = 0.459 to 0.921, *r* = − 0.142 to − 0.019; females: U = 24.00 to 47.00, *p* = 0.188 to 0.619, *r* = − 0.287 to − 0.108). Exceptions were found in lower-limb FM, where European-level judokas showed significantly greater inter-limb asymmetry compared to national-level judokas (males: U = 38.500, *p* = 0.015, *r* = − 0.470; females: U = 24.000, *p* = 0.033, *r* = − 0.465), and in lower-limb BMC, where female national-level judokas exhibited higher asymmetry than those at the European level (U = 25.000, *p* = 0.039, *r* = − 0.450).

**Table 3:** The magnitude of morphological inter-limb asymmetry (mean ± standard deviation) in male and female judokas across competitive levels (European and National level judokas)

	European-level	National-level	U-value	*p*-value	*r*-value
**Males (n)**	16	11			
LM - UL (%)	4.26 ± 2.74	4.91 ± 3.83	86.00	0.921	−0.019
LM - LL (%)	3.43 ± 2.37	2.90 ± 1.21	78.00	0.622	−0.095
BMD – UL (%)	5.12 ± 3.13	4.89 ± 4.09	77.00	0.587	−0.105
BMD – LL (%)	2.70 ± 1.86	2.26 ± 1.88	76.00	0.554	−0.114
BMC - UL (%)	6.74 ± 3.50	7.86 ± 5.52	78.00	0.622	−0.095
BMC - LL (%)	2.10 ± 1.81	2.21 ± 1.36	73.00	0.459	−0.142
FM – UL (%)	8.24 ± 5.51	7.72 ± 4.42	82.00	0.767	−0.057
FM – LL (%)	6.12 ± 4.53	2.70 ± 1.88	38.50	**0.015**	**-**0.470
**Females (n)**	12	9			
LM - UL (%)	4.64 ± 4.08	5.48 ± 4.07	45.00	0.522	−0.140
LM - LL (%)	3.90 ± 2.16	2.72 ± 1.85	35.50	0.188	−0.287
BMD – UL (%)	5.60 ± 2.66	5.77 ± 3.77	46.00	0.570	−0.124
BMD – LL (%)	1.99 ± 1.47	1.47 ± 0.84	47.00	0.619	−0.108
BMC - UL (%)	6.50 ± 2.50	6.93 ± 4.83	45.00	0.522	−0.140
BMC - LL (%)	1.53 ± 1.09	3.18 ± 1.98	25.00	**0.039**	−0.450
FM – UL (%)	5.30 ± 4.98	7.61 ± 6.46	39.00	0.286	−0.232
FM – LL (%)	3.71 ± 3.60	6.40 ± 2.79	24.00	**0.033**	−0.465

*U* Mann-Whitney U value, *p* statistical significance, *r* r-value, effect size, *UL * upper-limb, *LL* lower-limb, *LM* lean mass, *BMD* bone mineral density, *BMC* bone mineral content, *FM* fat mass

*p* < 0.05 indicates a significant difference in the magnitude of inter-limb asymmetry between European- and national-level judokas

Table 4 displays all sex-specific asymmetry magnitudes. There were no statistically significant differences in asymmetry magnitude between male and female judokas (U = 208.50 to 278.50, *p* = 0.119 to 0.975, *r* = −0.138 to −0.004).

**Table 4:** Inter-limb morphological asymmetry and differences between male and female judokas

	Male (*N* = 27)	Female (*N* = 21)	U-value	*p*-value	*r*-value
LM - UL (%)	4.53 ± 3.17	5.00 ± 4.00	274.00	0.843	−0.028
LM - LL (%)	3.21 ± 1.97	3.40 ± 2.07	272.00	0.811	−0.035
BMD – UL (%)	5.02 ± 3.48	5.67 ± 3.10	237.50	0.339	−0.138
BMD – LL (%)	2.52 ± 1.84	1.77 ± 1.24	208.50	0.119	0.225
BMC - UL (%)	7.20 ± 4.37	6.69 ± 3.58	282.00	0.975	−0.004
BMC - LL (%)	2.15 ± 1.61	2.23 ± 1.71	278.50	0.917	−0.015
FM – UL (%)	8.03 ± 5.00	6.29 ± 5.63	222.00	0.201	−0.184
FM – LL (%)	4.72 ± 4.01	4.86 ± 3.48	264.00	0.685	−0.058

U Mann-Whitney U value, *p* statistical significance, *r* r-value, effect size, *UL* upper-limb, *LL* lower-limb, *LM* lean mass, *BMD* bone mineral density, *BMC* bone mineral content, *FM* fat mass

## Discussion

This study is one of the first DXA-based investigations on youth judokas. It also examined differences in inter-limb asymmetry based on competition levels and sex. The magnitude of inter-limb asymmetry ranged from 1.47% to 8.24% across different outcomes, competition levels, and sexes. Significant differences in DXA-derived measures between the dominant and non-dominant limb were found regardless of competition level and sex, except for upper-limb BMD in both male and female European-level judokas. Significant differences between European- and national-level judokas were observed only in FM for both sexes and in the lower-limb BMC for females. No significant differences in inter-limb asymmetry were observed between sexes.

Previous research has suggested that in adolescent judokas, the use of personal techniques (tokui-waza) may result in repetitive unilateral movements during training and competition, potentially contributing to the development of morphological asymmetries [20]. Similarly, the present study revealed significant differences between the dominant and non-dominant limbs in both the upper and lower body, with asymmetry magnitudes ranging from 1.47% to 8.24%. Although these values are lower than those typically reported in predominantly unilateral sports such as tennis (5.5–17.6% in the upper limbs) [2], they are comparable to asymmetry levels observed in a general population of physical education students [24]. While judo involves inherently asymmetrical actions, elite judokas are typically trained to develop proficiency on both sides, as bilateral technical skill is essential for success in the sport [18, 19, 30]. This may explain why the asymmetry magnitudes were generally low and fell below the commonly reported, though arbitrary, thresholds of 10–15% that are often associated with performance deficits or injury risk [3, 31].

As shown by the differences in raw segmental DXA data (Table 3), increased judo practice can lead to changes in body composition particularly in LM, BMD and BMC. Compared with both endurance sports and other impact sports such as tennis, judokas display substantially higher LM, BMD, and BMC values [32, 33]. Longitudinal research further supports that judo training during childhood and adolescence provides multiple health benefits, including improvements in cardiovascular function, a more mesomorphic somatotype, and enhanced bone health [34, 35]. Nevertheless, it is important to note that during growth, athletes are particularly susceptible to injuries, which are often closely linked to inappropriate training loads during periods of peak height velocity [36]. Therefore, practitioners should be aware that large changes in body composition among youth judokas should be carefully monitored and managed. Implementing bio-banding strategies, where athletes are grouped according to their biological and maturational stage rather than chronological age, may help ensure appropriate training loads and reduce injury risk [36].

Growth is not a linear process, and the rate of change may vary across different body segments [37]. However, our analysis revealed minimal differences in inter-limb asymmetry between national- and European-level judokas. Although increased training exposure has been identified as an important determinant of judo-specific performance [38], the present findings suggest that distinguishing competitive levels based on morphological inter-limb asymmetry profiles is challenging. Significant differences were observed only in FM (for both sexes) and BMC (only in female judokas). This finding partially contrasts with previous research, which reported no significant differences in inter-limb morphological asymmetry between competitive levels [12, 14]. Notably, our study revealed that FM asymmetry was not consistent across sexes, being higher in European-level male judokas but lower in European-level females. While true sex differences may account for this, it is also important to consider that the DXA precision error for FM can reach up to 2.8% at the segmental level, which may contribute to the observed variability [39]. Moreover, since segmental FM distribution is not easily modified through training [40], these differences likely have limited practical relevance. Therefore, practitioners are advised to aim to maintain or achieve low whole-body FM rather than focusing on side-to-side differences, as FM represents non-active metabolic tissue that can impair judo performance [41, 42].

Furthermore, our findings showed that BMC asymmetry was significantly greater in national-level female judokas compared to the European-level peers. Given that bone tissue remodels in response to mechanical stress through mechanotransduction, it is possible that segmental BMC in judokas is highly dependent on judo-specific loading over time [43]. Therefore, we hypothesize that a greater emphasis on bilateral training among European-level judokas compared to national-level judokas may explain these differences [18, 35].

Although body composition clearly differs by sex in judokas [20], our findings align with previous research showing no significant differences in inter-limb morphological asymmetry across sexes [14, 44]. Similar results have been reported for functional asymmetry, with no significant differences in inter- or intra-limb isokinetic strength asymmetries observed among world-level, European-level, and national-level judokas for most asymmetry indices [11]. These findings support the notion that morphological and functional differences between sexes are more pronounced at the whole-body level than at the segmental level. Consequently, sex does not appear to be a major determinant of inter-limb asymmetries. This underscores the importance of adopting an individualized approach when monitoring and interpreting asymmetries.

The present study is not without limitations. First, maturity status was not assessed in this study. Previous research has reported that the maturity status as well as the relative age effect are important determinants of judo performance in youth judokas [38, 44]. Additionally, evidence suggests that the maturation process contributes to injury risk [26]. Given that morphological inter-limb asymmetry has also been reported to be influenced by maturity status, future studies should consider examining the impact of maturation in their analysis using a longitudinal design [25]. Second, the athletes’ training history was not recorded, which could also explain their (segmental) morphological body profiles [45]. However, all participants held at least a brown or black belt, which generally reflects a minimum of three to five years of structured judo practice. Finally, although comparisons were made across competition levels, the cross-sectional design precludes causal inference regarding the relationship between training exposure and asymmetry development. Future longitudinal studies are needed to clarify these potential associations.

## Conclusion

In conclusion, the observed differences between the dominant and non-dominant upper and lower limbs in youth judokas, ranging from 1.47% to 8.24%, indicate relatively low levels of inter-limb asymmetry. Therefore, addressing these asymmetries should not be considered a primary focus in training or performance optimization. The limited differences across competition levels and the absence of significant sex-based differences suggest that no specific group is at greater risk of performance decline or injury due to asymmetry. These findings support the adoption of an individualized approach to monitoring and managing inter-limb asymmetries, rather than applying uniform interventions across athlete groups. Moreover, coaches and practitioners should focus on the long-term monitoring of asymmetry development throughout growth and training cycles. Future research should examine the causal relationship between the magnitude of inter-limb asymmetry and performance decrements or injury risk.

## Data Availability

The datasets generated and/or analysed during the current study are available in the Vrije Universiteit Brussel repository, with persistent identifier VUB/MOVE/1/000005.

## Abbreviations

DXA: Dual-energy X-ray Absorptiometry
BIA: bioelectrical impedance analysis
FM: fat mass
BMD: bone mineral density
BMC: bone mineral content
LM: lean mass
BMI: body mass index
